# ROMPI-CDSA: ring-opening metathesis polymerization-induced crystallization-driven self-assembly of metallo-block copolymers†
†Electronic supplementary information (ESI) available. See DOI: 10.1039/c9sc03056e


**DOI:** 10.1039/c9sc03056e

**Published:** 2019-09-04

**Authors:** Ye Sha, Md Anisur Rahman, Tianyu Zhu, Yujin Cha, C. Wayne McAlister, Chuanbing Tang

**Affiliations:** a Department of Chemistry and Biochemistry , University of South Carolina , Columbia , South Carolina 29208 , USA . Email: tang4@mailbox.sc.edu

## Abstract

Polymerization-induced self-assembly (PISA) and crystallization-driven self-assembly (CDSA) are among the most prevailing methods for block copolymer self-assembly. Taking the merits of scalability of PISA and dimension control of CDSA, we report one-pot synchronous PISA and CDSA *via* ring-opening metathesis polymerization (ROMP) to prepare nano-objects based on a crystalline poly(ruthenocene) motif. We denote this self-assembly methodology as ROMPI-CDSA to enable a simple, yet robust approach for the preparation of functional nanomaterials.

## Abstract 2

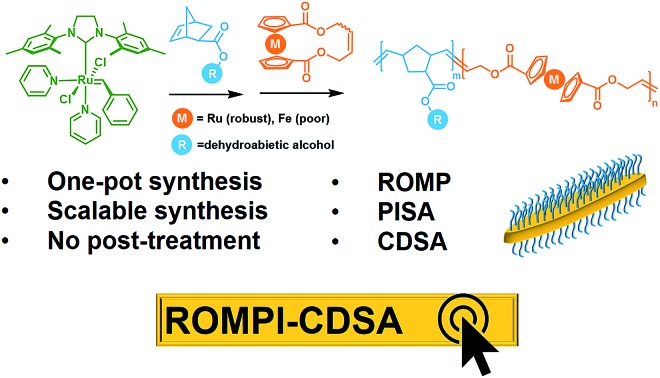
Two most prevailing self-assembly methods, PISA and CDSA, are combined in one metallo-block copolymer system *via* one-pot synchronous ROMP, yielding crystalline nanostructures in a mild, fast, scalable and controlled manner.

## Introduction

Block copolymer (BCP) self-assembly in solution provides a versatile tool to build a wide variety of micellar morphologies, ranging from spheres to vesicles to many other complex structures.^1^–^8^ Notwithstanding significant advances, the preparation of BCP micelles is usually a protracted multi-step process, which typically includes precise sequential polymerization, tedious purification and sophisticated assembly steps, making it formidable for scalability.^9^–^12^

Scalable processing (under concentrated conditions, typically 5–50% w/w solids) of well-defined micelles can be realized by an approach termed polymerization-induced self-assembly (PISA) where the polymerization of BCPs and self-assembly take place *in situ*.^10^,^13^–^21^ However, the preparation of fiber-like or platelet micelles with uniformity is challenging to access from the self-assembly of non-crystalline BCPs.^22^ This has led to the development of crystallization-driven self-assembly (CDSA) of BCPs where the crystalline core largely dictates the formation of 1D and 2D nanostructures.^1^,^23^–^28^ Nevertheless, multiple steps of synthesis and purification together with very low concentration solution processing (typically <0.5% w/w solids) severely limit the scalability of CDSA.^29^

Taking advantage of the scalability and dimensional control respectively from PISA and CDSA, a new process called polymerization-induced CDSA (or PI-CDSA) would be promising, but challenging.^22^,^29^ PISA is predominantly conducted *via* living radical polymerization technologies, where reversible addition–fragmentation chain transfer (RAFT) polymerization prevails.^10^ However, radical polymerization usually leads to atactic polymers, which are not preferred for crystallization. Recently, Manners and his co-workers reported the first and so far the only example of PI-CDSA by using unique properties of poly(ferrocenyldimethylsilane) *via* living anionic polymerization under stringent conditions.^22^,^29^ This unparalleled paradigm has inspired us to question whether this approach can be applicable to more mild polymerization methods and other crystalline polymers. Ring-opening metathesis polymerization (ROMP) has shown the potential in PISA due to its access to different classes of monomers.^12^,^30^,^31^ Additionally, highly conjugated crystalline polymer species can be also prepared *via* ROMP.^32^,^33^

In this work, we report ROMP as a new polymerization method to achieve the PI-CDSA with ruthenocene-containing polymers as new crystallizable species. It is termed as ring-opening metathesis polymerization-induced crystallization-driven self-assembly (ROMPI-CDSA). Platelet hybrid nanostructures were obtained using this one-pot process without any post-treatment. The crystalline polyruthenocene core segment is an exceptional driving force to dictate the self-assembly. The work represents new efforts toward emerging directions of metallopolymers for advanced materials by us and others.^34^–^45^

## Results and discussion

The synchronous process of PI-CDSA requires the incipient production of a corona-forming block that is carried out under living polymerization in a good solvent medium. The same solvent system becomes non-selective for the second core-forming block as the degree of polymerization (DP) of the second segment proceeds to a threshold that induces crystallization.^22^

To meet this criteria, we first targeted a block copolymer poly(5-methoxycyclooctene)-*b*-polyferrocene (PMCOE-*b*-PFc) in tetrahydrofuran (THF) (Scheme S1†), as THF is a good solvent for PMCOE but poor for PFc, and the CDSA behavior of this BCP in THF was recently demonstrated by us.^46^ However, this seemingly straightforward process proved nontrivial. After the complete consumption of 5-methoxycyclooctene monomer to form the corona block, cyclic ferrocenyl olefin monomer was added into the polymerization system to achieve a final concentration of 10% w/w solids, which is within the typical concentration regime of PISA but well above the concentration for CDSA (<0.5% wt).^22^ It was anticipated that the PFc block would become insoluble and precipitate from the solution when its DP reaches a threshold where ROMPI-CDSA might occur. As shown in Table S1,† initial experiments with a wide range of block ratios from 1 : 0.1 to 1 : 1 in the polymerization system were carried out. When the conversion of second monomer reached the upper limit, ethyl vinyl ether (EVE) was added to quench the polymerization, and small aliquots of the solutions were diluted by THF and then cast onto copper grids for transmission electron microscopy (TEM) analysis. Only non-crystalline spheres or irregular aggregations were observed for these compositions (Fig. S9†). One possible reason is that the target molecular weight might be too high (over 100 000 Da) to form non-spherical morphologies,^47^ and the rate of crystallization for the PFc core-forming block would decrease when its chain length increases.^48^ We then decreased the target molecular weight to 45 000 Da with a wide range of block ratios (Table S2†). However, spheres or irregular aggregations were still observed, as shown in Fig. S10.† The ROMP process to prepare polycyclooctene is generally not a living process,^49^ thus it may lead to ineffective initiation and growth of the core-forming block, which could make the architecture of diblock copolymers in an ill-defined manner.

**Fig. 1:**
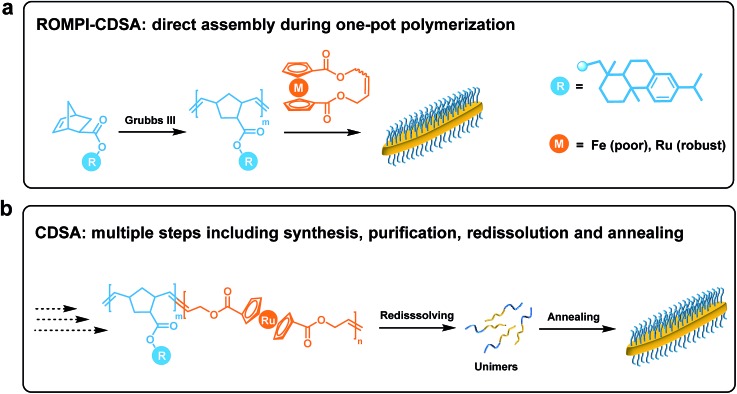
Schematic comparison between (a) ROMPI-CDSA process that allows simple, direct assembly of block copolymers during one-pot synthesis, and (b) CDSA process that involves multiple steps including synthesis, purification, redissolution and annealing.

It is well established that norbornene-based strained olefins undergo living ROMP efficiently.^50^–^53^ Since pristine polynorbornene can be crystalline,^54^ we grafted a bulky tricyclic group (dehydroabietic) as the side-chain moiety onto *exo*-5-norbornenecarboxylic acid to not only increase the solubility of the corona-forming block but also elevate its glass transition temperature (∼110 °C) to facilitate assembled morphologies unaffected under room temperature.^52^ As shown in Fig. 1, main-chain ferrocene-containing diblock copolymers can be prepared by sequential ROMP. Narrow molecular weight distribution (Fig. S6†) and predicted molecular weight indicated the living manner of the first block of polynorbornene (PNR), the yielded BCPs with a series of segment compositions are given in Table 1. TEM measurements showed the formation of fiber-like morphologies, demonstrating that self-assembly took place during the polymerization process (Fig. S11†). However, these micelles are surrounded by pronounced background, which can be attributed to the low crystallinity of PFc prepared by ROMP.^46^ It has been demonstrated that higher crystallinity is required to achieve successful CDSA.^46^,^55^,^56^ Based on these results, it was suggested that PFc-based BCPs are not sufficient to produce well-defined nanostructures *via* the ROMPI-CDSA process, but inspire further exploration on other metallocene-based BCPs with higher crystallinity.

**Table 1:** Metallocene-containing BCPs used for ROMPI-CDSA

Sample	Block copolymer^*a*^	*M*_n_ ^*b*^ (Da)
1	PNR_123_-*b*-PFc_93_	80 300
2	PNR_123_-*b*-PFc_36_	618 00
3	PNR_52_-*b*-PFc_71_	44 500
4	PNR_52_-*b*-PFc_43_	35 400
5	PNR_31_-*b*-PRc_49_	30 800
6	PNR_31_-*b*-PRc_21_	20 400
7	PNR_48_-*b*-PRc_56_	40 300

^*a*^The DP of the first block was determined from GPC, the DP of the second block was determined from ^1^H NMR *via* compositional analysis.

^*b*^The number-average molecular weight was calculated based on the DP of the diblock copolymer.

**Fig. 2:**
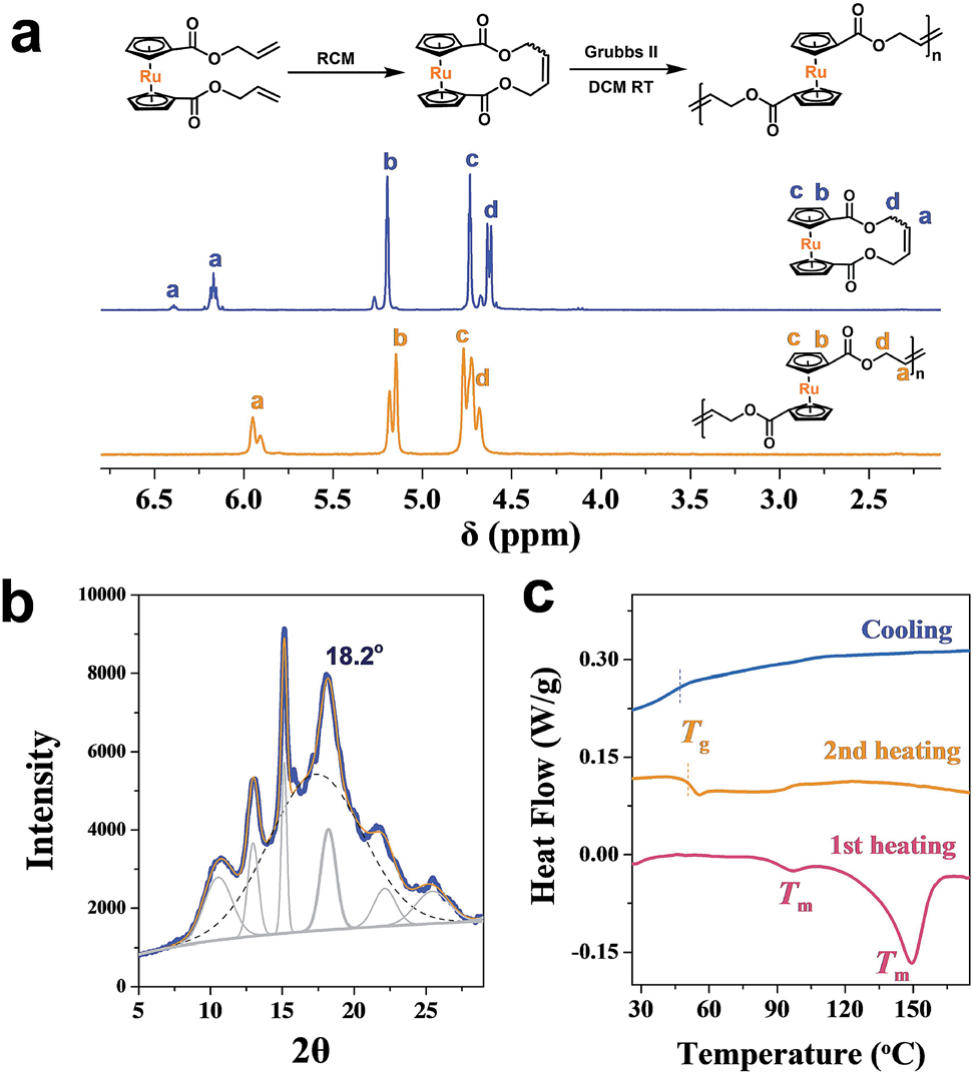
(a) ^1^H NMR spectra (CDCl_3_) of cyclic ruthenocene olefin and main-chain ruthenocene-based homopolymer; (b) XRD powder spectrum of PRc homopolymer; (c) DSC curves of PRc.

As an analogy to ferrocene, ruthenocene-based polymers are somehow much less explored.^57^–^64^ Using a similar methodology,^46^,^64^,^65^ a cyclic ruthenocenyl olefin monomer was prepared (Fig. 2a). Interestingly, polyruthenocene (PRc) is crystalline as confirmed by X-ray Diffraction (XRD, Fig. 2b and S1†) and Differential Scanning Calorimetry (DSC, a melting transition observed during the heating scan, Fig. 2c and S3†). Surprisingly, it has much higher crystallinity (∼38.3%) than PFc (∼17.4%). It might be due to the presence of the *trans*-dominated conformation of carbon–carbon double bonds along the backbone (as indicated by solid-state ^13^C NMR spectra, Fig. S4†), which induces higher crystallization.^66^,^67^ It is thus intriguing to explore ROMPI-CDSA for ruthenocene-containing polymers.

**Fig. 3:**
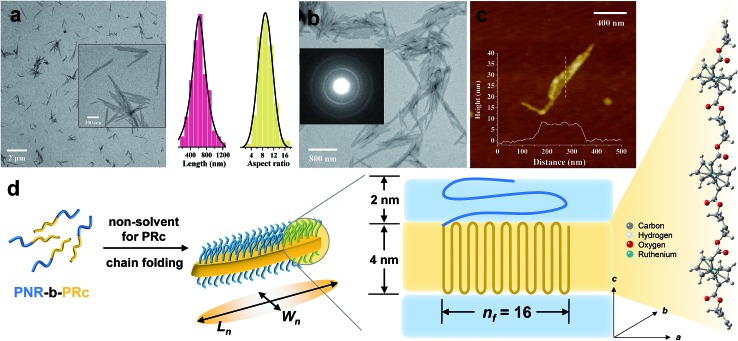
(a) Low resolution TEM image of PNR_31_-*b*-PRc_49_ micelles *via* ROMPI-CDSA; the inset shows the high resolution image; the statistical plots show data of lengths and aspect ratios; (b) high-resolution TEM image of PNR_31_-*b*-PRc_49_ micelles formed after polymerization, the inset image shows the SAED pattern; (c) AFM height image and profile of PNR_31_-*b*-PRc_49_ micelles; the height profile is from the indicated dash line; (d) a schematic diagram illustrating the chain folding of the crystalline PRc core. The stick-ball structure indicates the possible alignment of PRc segment along the perpendicular direction.

Following the same procedure as ROMPI-CDSA of the ferrocene monomer, ruthenocene-containing block copolymer PNR-*b*-PRc was prepared with different block ratios (Table 1). TEM measurements of diluted PNR_31_-*b*-PRc_49_ samples showed exclusive lenticular platelet structures with a number-average length of *L*_n_ = 645 nm and a low dispersity of 1.10 (Fig. 3a). The aspect ratio (*L*_n_/*W*_n_, where *W*_n_ is the number-average width) was statistically analyzed as 9.5 with a low dispersity of 1.09 (Fig. 3a). Selected-area electron diffraction (SAED) pattern of TEM (Fig. 3b) showed a well-defined diffraction ring with *d* = 4.9 Å, indicating the crystalline nature of PRc core. This result confirms that the self-assembly process was driven by the crystallization of PRc core, as suggested in related to the distance calculated from Fig. 2b. AFM height image indicated that the height of platelets is approx. 8 nm (Fig. 3c). The crystalline core of PRc is expected to be sandwiched between the PNR coronas (Fig. 3d). The thickness of crystalline domains was estimated to be 4 nm (see ESI† for analysis).^68^,^69^ Together with the estimated contour length of crystalline chain, the PRc segment (DP = 49) is folded *ca.* 16 times (*n*_f_ = 16, Fig. 3d), considering the proximity of chain packing in the direction normal to the fold surface.^70^

In a separate experiment, this BCP was isolated and purified for studying the CDSA process as illustrated in Fig. 1b. This standalone process resulted in the formation of similar platelet structures (Fig. S13†). This additional experiment demonstrated that the one-pot synthesis of nanostructures was largely driven by crystallization of the PRc core. ROMPI-CDSA of other BCPs (PNR_31_-*b*-PRc_21_ and PNR_48_-*b*-PRc_56_) also produced lenticular platelets, as shown in Fig. S14 and S15.† The decrease in platelet width of PNR_31_-*b*-PRc_21_ compared to PNR_31_-*b*-PRc_49_ is in a good agreement with the decreased chain length of the crystalline PRc segment.

**Fig. 4:**
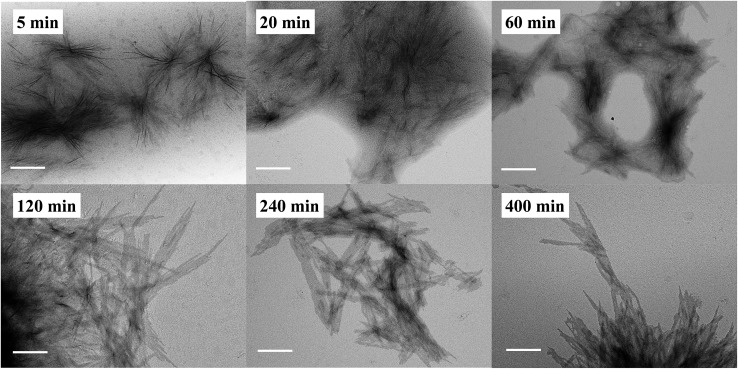
Representative higher resolution TEM images of PNR_48_-*b*-PRc_*n*_ micelles by ROMPI-CDSA with aliquots taken out at specific time intervals. The scale bar in each image is 400 nm. *n* is the DP of PRc.

In addition to the CDSA process, it is equally important to determine if the polymerization and self-assembly took place simultaneously, *i.e.*, whether is it only a PISA process? During the ROMPI-CDSA process to target a BCP of PNR_48_-*b*-PRc_56_, aliquots were taken out at specified time intervals for ^1^H NMR and TEM characterization to provide mechanistic insights into the self-assembly. The diblock copolymer composition was summarized in Table S3.† As shown in Fig. 4 (higher resolution) and Fig. S16† (lower resolution), no micelles were observed during the first minute with a DP = 9 of PRc. At 5 min, ill-defined fiber-like features were observed. At 20 min, large quantities of aggregated fibers appeared, and platelet structures started to form. When polymerization went for 60 min with DP = 33, platelet structures became more pronounced. At time 120 min with a DP = 38 of PRc, a morphological transition occurred with dominated platelet micelles present and almost without fiber-like micelles. When time reached over 240 min, exclusive platelet micelles were observed. A test on the Tyndall effect of reaction solutions could provide additional evidence on the PISA process, simply by projecting a laser beam through the reaction vials. It clearly showed the increasing Tyndall effect with evolution of reaction time, until the formation of visibly turbid solutions (Fig. S17†). It was then concluded that ROMP and self-assembly occurred simultaneously through the ROMPI-CDSA process, corresponding to a morphological transformation from fibers at low DP to platelets at high DP.^22^,^71^–^73^

## Conclusions

In summary, we presented a new self-assembly process termed as ROMPI-CDSA. This one-pot synthesis combined synchronous ROMP, PISA and CDSA to yield uniform crystalline nano-objects in a mild, fast, scalable and controlled manner. The mechanistic insights by exploring ferrocene-containing BCPs towards ROMPI-CDSA unveiled the interplay of various parameters including molecular weight, chain extension and crystallinity. It was discovered that higher crystalline ruthenocene-containing BCPs self-assembled *via* the ROMPI-CDSA process to allow the scalable production of lenticular platelet nanomaterials at concentrations ∼10% w/w solids. The ROMPI-CDSA could be viewed as a new paradigm that is applicable to other BCP systems with a crystalline core-forming block, which can be prepared by mild polymerization techniques.

## Conflicts of interest

There are no conflicts to declare.

## Supplementary Material

Supplementary information
