# Supplementary material for: ROMPI-CDSA: ring-opening metathesis polymerization-induced crystallization-driven self-assembly of metallo-block copolymers
Source: Chem Sci. 2019 Sep 4;10(42):9782–7. doi: 10.1039/c9sc03056e (PMC6993615; doi:10.1039/c9sc03056e)
Supplement: Supplementary file 1 [file SC-010-C9SC03056E-s001.pdf]

Electronic Supplementary Material (ESI) for Chemical Science.
This journal is © The Royal Society of Chemistry 2019

Supplementary Information

ROMPI-CDSA: Ring-Opening Metathesis Polymerization Induced-Crystallization-Driven Self-Assembly of Metallo-Block Copolymers

Ye Sha, Md Anisur Rahman, Tianyu Zhu, Yujin Cha, C. Wayne McAlister and Chuanbing Tang

Department of Chemistry and Biochemistry, University of South Carolina, Columbia, South Carolina 29208, United States

1. General Information 1
  1.1 Materials 1
  1.2 Characterization and sample preparation 2
2. Synthesis of ruthenocene-based homopolymers 2
  2.1 Synthesis 2
  2.2 Characterization 3
3. Synthesis and characterization of diblock copolymers 6
  3.1 Synthesis of PMCOE-*b*-PFc 6
  3.2 Characterization of PMCOE-*b*-PFc 8
  3.3 Synthesis of PNR-*b*-PFc 10
  3.4 Characterization of PNR-*b*-PFc 11
  3.5 Synthesis and PNR-*b*-PRc 12
  3.6 Characterization of PNR-*b*-PRc 14
4. ROMPI-CDSA of PMCOE-*b*-PFc 15
5. ROMPI-CDSA of PNR-*b*-PFc 16
6. Comparison of XRD spectrum for RPc homopolymers and block copolymers 16
7. CDSA behavior of PNR_{31}-*b*-PRc_{49} 17
8. Chain folding analysis of PNR_{31}-*b*-PRc_{49} micelles prepared via ROMPI-CDSA 17
9. ROMPI-CDSA of PNR_{31}-*b*-PRc_{21} 18
10. ROMPI-CDSA of PNR_{48}-*b*-PRc_{56} 18
11. ROMPI-CDSA of PNR_{48}-*b*-PRc_{n} 19
12. References 20

1. General Information

1.1 Materials

Ethyl vinyl ether (EVE, 99%) and Grubbs II catalyst (98%) were purchased from Sigma-Aldrich and used as received. Grubbs III catalyst was prepared according to literature.[1] 5-Methoxycyclooctene, 1,1'-(2-butenyl)ferrocenedicarboxylate and 1,1'-

(2-butenyl)ruthenocenedicarboxylate were prepared based on our previous work.[2] Dehydroabietic alcohol modified norbornene was prepared based on our previous work.[3] All solvents were dried and freshly distilled before use. All synthetic procedures were carried out under nitrogen protection.

1.2 Characterization and sample preparation

^{1}H NMR spectra were recorded on a 300 MHz Bruker NMR using $CDCl_3$ as solvent with chemical shifts reported with respect to $CHCl_3/CDCl_3$ ($\delta(^1H)$ =7.26 ppm). Solid-state ^{13}C NMR experiments were performed in 500 MHz Bruker NMR using a cross-polarization magic-angle spin (CP-MAS) method. Gel permeation chromatography (GPC) was performed using HPLC-grade tetrahydrofuran (THF) as eluent with a flow rate of 1 mL/min at 35 °C on a Waters-GPC equipment. It was installed with a refractive index (RI) detector with narrow dispersed polystyrene (PS) as the molecular weight standard. TEM images were collected from a JEOL 1400 Plus Transmission Electron Microscope with an accelerating voltage of 120 kV. Diffraction images were collected on a diffraction accessory of JEOL 1400 Plus Transmission Electron Microscope. The samples for electron microscopy were prepared by drop casting one drop of sample solution (ca. 0.5 mg/mL) onto a copper grid and fully dried at room temperature before measurement. Powder X-ray diffraction (XRD) spectra were collected on a Rigaku D/Max 2100 Powder X-Ray Diffractometer instrument (Cu-Kα radiation) scanning from 5° to 30° with a step size of 0.04° and a step rate of 2 s. Atomic force microscopy (AFM) images were collected from a Bruker Multimode Nanoscope V system based on a tapping mode using an oscillating tip. The measurements were conducted using commercial silicon cantilevers with a nominal spring constant at 20-80 N/m and a resonance frequency at 230-410 kHz. The samples for AFM were prepared by drop casting one drop of sample solution (0.5 mg/mL) onto a silicon wafer and fully dried at room temperature before measurement. DSC measurements was conducted on a Mettler-Toledo DSC1 DSC instrument using indium and zinc as internal references under N_2 condition (50 mL/min), the heating and cooling rate was fixed at 10 °C/min.

2. Synthesis of ruthenocene-based homopolymers

2.1 Synthesis

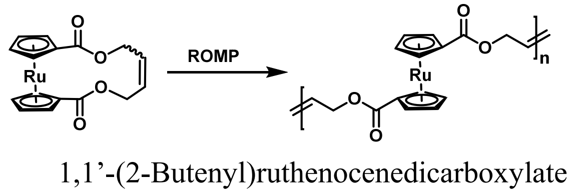

1,1'-(2-Butenyl)ruthenocenedicarboxylate (0.6463 mmol, 240 mg, 100 eq) was dissolved in 4.8 mL dry DCM. Then Grubbs II catalyst (0.006463 mmol, 5.49 mg, 1 eq) dissolved in 0.2 mL DCM was injected into the system to initiate ROMP. During the polymerization, the polymer precipitated from the solution. The reaction was quenched by adding one drop of EVE after reacting for 10 h (conversion = 89% determined from

^{1}H NMR). The insoluble polymer was dispersed in DCM and precipitated into methanol for three times and dried at room temperature, yielding 200 mg product.

2.2 Characterization

2.2.1 XRD analysis

Based on the full width at half-maximum height of the main peak from the powder XRD spectrum of PRc at 18.2° (Fig. 2a), the crystallite size of 71.2 Å calculated from the Scherrer equation:

$$L_{hkl} = \frac{1}{\frac{2\cos\theta}{\lambda}\delta\theta} = \frac{1}{\frac{2\cos(18.2/2)}{1.5406} * \frac{1.2557/2}{180} * 3.14} = 71.2$$

The crystallinity can be estimated by the deconvolution of XRD profile, sharp peaks represent crystalline regions whereas the broad peak represents the non-crystalline region. The crystallinity was calculated to be 38.3% based on fraction of areas under crystalline peaks in the total area under both non-crystalline and crystalline regions. For PFc, the crystallinity was estimated to be around 17.4%. As Figure S1 indicates, PRc shows much higher crystallinity than PFc.

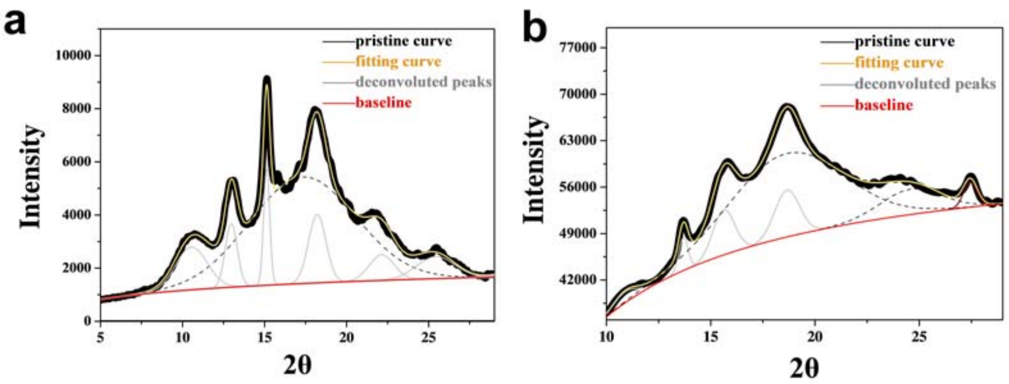

Figure S1. Powder XRD spectra of (a) PRc and (b) PFc. The powder samples were directly characterized after polymerization without thermal treatment. Dashed lines indicate scattering of materials from amorphous phase.

2.2.2 Thermal analysis

A typical thermal analysis protocol was set up as shown in Figure S2a. The first heating curve of polymers directly obtained after polymerization shows a dominant melting peak at 149.3 °C and a minor melting peak at 96.2 °C. Then cooling at a rate of 10 °C/min is rapid enough to quench the melt, resulting non-crystalline polymers. Subsequent heating at 10 °C/min shows the glass transition temperature of 49 °C. The first heating scan around 49 °C shows no apparent T_g, indicating that the obtained polymer after polymerization is crystalline. Additionally, the glass transition should be very pronounced during the first heating scan since the sample has been already annealed at room temperature overnight, resulting in pronounced enthalpy relaxation

overshoot if the polymer is amorphous but the heating scan proves none observable T_g. The T_g transition appears in the second heating, in turn demonstrating that this pronounced glass transition region is originated from the domain where polymer crystal melts. Isothermal crystallization is helpful for nucleation. Two typical annealing processes were attempted at 90 °C and 110 °C for 30 min, respectively. However, it is still hard to recover crystalline PRc again by thermal treatment. It should be noted that the heat capacity keeps unchanged during two scans, indicating that the polymer is stable during these scans. The mechanism of polymerization-induced crystallization in solution and thermal treatment-facilitated crystallization can be different because the microenvironment for polymer chain folding is different in bulk and solution. An exhaustive crystallization kinetics deserves a separated study in the future. In any case, the thermal analysis results indicate that the PRc homopolymer obtained from polymerization is crystalline.

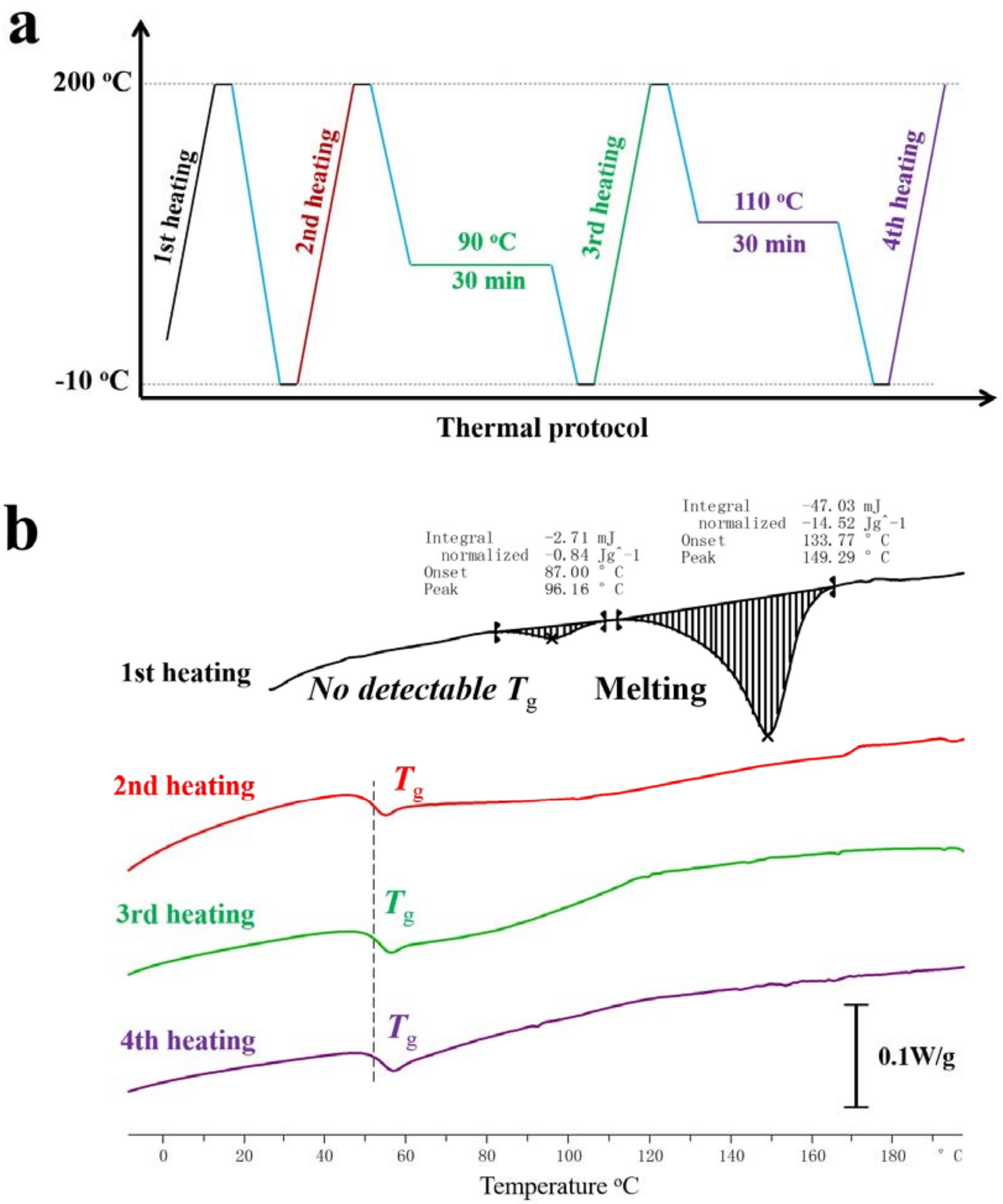

Figure S2. (a) Thermal protocols for thermal analysis for PRc; (b) DCS traces of a PRc homopolymer. Both heating and cooling rates were 10 °C /min.

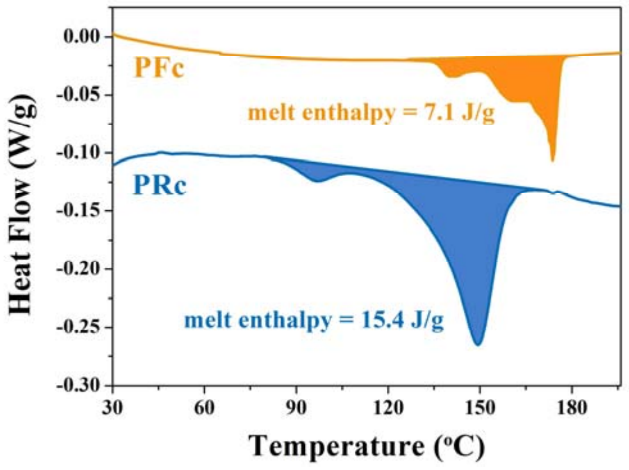

Figure S3. First heating DSC traces of PFc and PRc homopolymers after polymerization. Both heating rates were 10 °C/min.

2.2.3 Solid-state ^{13}C NMR spectra

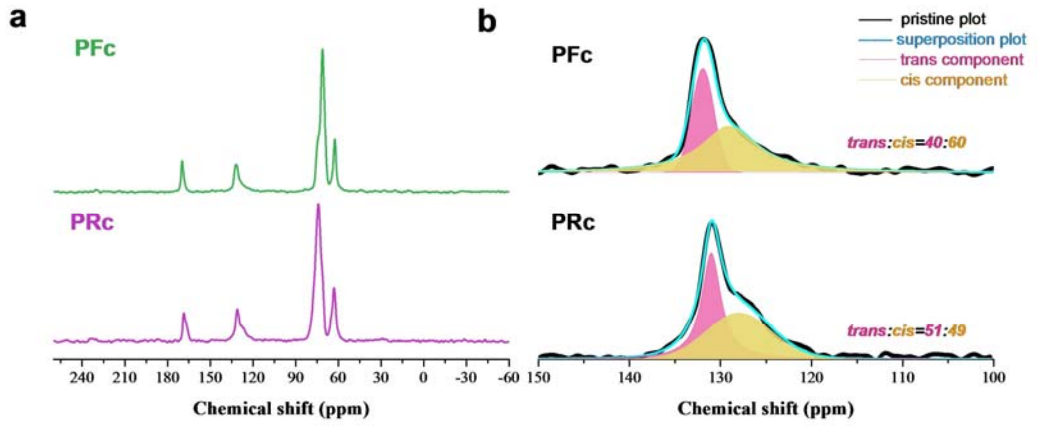

Figure S4. (a) Solid-state ^{13}C NMR spectra of PFc and PRc homopolymers; (b) ^{13}C NMR peak deconvolution of carbon-carbon double bond.

3. Synthesis and characterization of diblock copolymers

3.1 Synthesis of PMCOE-*b*-PFc

Scheme S1. Synthesis of block copolymer PMCOE-*b*-PFc.

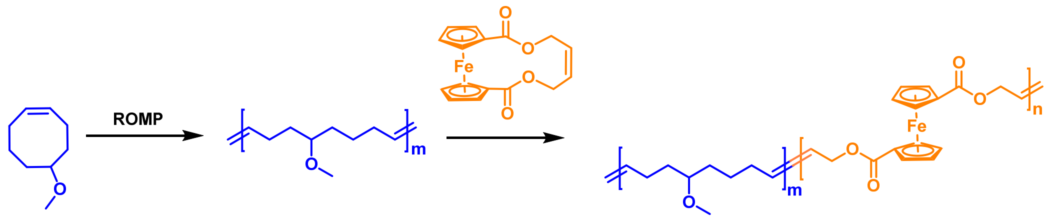

3.1.1 Synthesis of PMCOE$_{471}$-*b*-PFc$_{52}$

5-Methoxycyclooctene (86 mg, 0.6133 mmol, 500 eq) was dissolved in 0.9 mL dry THF, then Grubbs II catalyst (1.041 mg, 0.001227 mmol, 1 eq) dissolved in 0.25 mL THF was added into the above mixture and reacted for 16 min at room temperature for complete consumption of 5-methoxycyclooctene (0.25 mL solution was removed from the solution for ^{1}H NMR and GPC analysis, M_n = 66,000 Da, $Đ$ = 1.57). Then 1,1'-(2-butenyl)ferrocenedicarboxylate (0.06133 mmol, 20 mg, 50 eq) dissolved in 0.05 mL THF was added into the residual 1 mL solution and reacted for another 100 min to reach a conversion of 88%. The reaction was quenched using EVE. The resultant polymer can be purified by dissolving in chloroform and precipitating into methanol three times and dried, yielding ~ 70 mg solid.

3.1.2 Synthesis of PMCOE$_{471}$-*b*-PFc$_{104}$

5-Methoxycyclooctene (86 mg, 0.6133 mmol, 500 eq) was dissolved in 0.9 mL dry THF, then Grubbs II catalyst (1.041 mg, 0.001227 mmol, 1 eq) dissolved in 0.25 mL THF was added into the above mixture and reacted for 16 min at room temperature for complete consumption of 5-methoxycyclooctene (0.25 mL solution was removed from the solution for ^{1}H NMR and GPC analysis). 1,1'-(2-Butenyl)ferrocenedicarboxylate (0.1227 mmol, 40 mg, 100 eq) dissolved in 0.1 mL THF was added into the residual 1 mL solution and reacted for 10 h to reach a conversion of 81%, then the reaction was quenched using EVE. The resultant polymer was dispersed in chloroform, precipitated into methanol for three times, and dried, yielding ~ 85 mg solid.

3.1.3 Synthesis of PMCOE$_{471}$-*b*-PFc$_{245}$

5-Methoxycyclooctene (86 mg, 0.6133 mmol, 500 eq) was dissolved in 0.9 mL dry THF, then Grubbs II catalyst (1.041 mg, 0.001227 mmol, 1 eq) dissolved in 0.25 mL THF was added into the above mixture and reacted for 16 min at room temperature for complete consumption of 5-methoxycyclooctene (0.25 mL solution was removed from the solution for ^{1}H NMR and GPC analysis), then 1,1'-(2-butenyl)ferrocenedicarboxylate (0.3067 mmol, 100 mg, 250 eq) dissolved in 0.25 mL THF was added into the residual 1 mL solution and reacted for 10 h to reach a conversion of 65%. The reaction was quenched using EVE. The resultant polymer was dispersed in chloroform, precipitated into methanol for three times, and dried, yielding ~ 110 mg solid.

3.1.4 Synthesis of PMCOE$_{471}$-*b*-PFc$_{369}$

5-Methoxycyclooctene (86 mg, 0.6133 mmol, 500 eq) was dissolved in 0.9 mL dry THF, then Grubbs II catalyst (1.041 mg, 0.001227 mmol, 1 eq) dissolved in 0.25 mL THF was added into the above mixture and reacted for 16 min at room temperature for complete consumption of 5-methoxycyclooctene (0.25 mL solution was removed from

the solution for ^{1}H NMR and GPC analysis), then 1,1'-(2-butenyl)ferrocenedicarboxylate (0. 6133 mmol, 200 mg, 500 eq) dissolved in 0.5 mL THF was added into the residual 1 mL solution and reacted for 10 h to reach a conversion of 47%. The reaction was quenched using EVE. The resultant polymer was dispersed in chloroform, precipitated into methanol for three times, and dried, yielding ~ 140 mg solid.

3.1.5 Synthesis of PMCOE$_{92}$-*b*-PFc$_n$

5-Methoxycyclooctene (38.7 mg, 0.2760 mmol, 100 eq) was dissolved in 0.3 mL dry THF, then Grubbs II catalyst (2.343 mg, 0.002760 mmol, 1 eq) dissolved in 0.1 mL THF was added into the above mixture and reacted for 15 min at room temperature for complete consumption of 5-methoxycyclooctene (0.1 mL solution was removed from the solution f for ^{1}H NMR and GPC analysis, M_n =12,900 Da, $Đ$ = 1.61), then 1,1'-(2-butenyl)ferrocenedicarboxylate (0.1840 mmol, 60 mg, 66.7 eq) dissolved in 0.3 mL THF was added into the residual 0.2 mL solution to grow the second block, some aliquots of sample was removed at specified time intervals and quenched using EVE, then diluted for TEM measurements and ^{1}H NMR analysis to determine the conversion. The characterization data are summarized in Table S2.

3.2 Characterization of PMCOE-*b*-PFc

Table S1. Ferrocene-containing diblock copolymers for ROMPI-CDSA study.

Sample	Target Block Ratio *m:n*	Obtained Block Ratio	conv[a]	BCP[b]	M_n (Da)[c]
1	10:1	10:1.1	88%	PMCOE$_{471}$-*b*-PFc$_{52}$	83,000
2	10:2	10:2.2	81%	PMCOE$_{471}$-*b*-PFc$_{104}$	100,000
3	10:5	10:5.2	65%	PMCOE$_{471}$-*b*-PFc$_{245}$	146,000
4	10:10	10:7.8	47%	PMCOE$_{471}$-*b*-PFc$_{369}$	186,400

[a]The conversion was determined from ^{1}H NMR analysis.

[b]The DP of the first block was determined from GPC, the DP of the second block was determined from ^{1}H NMR.

[c]The number molecular weight was calculated based on the DP of the diblock.

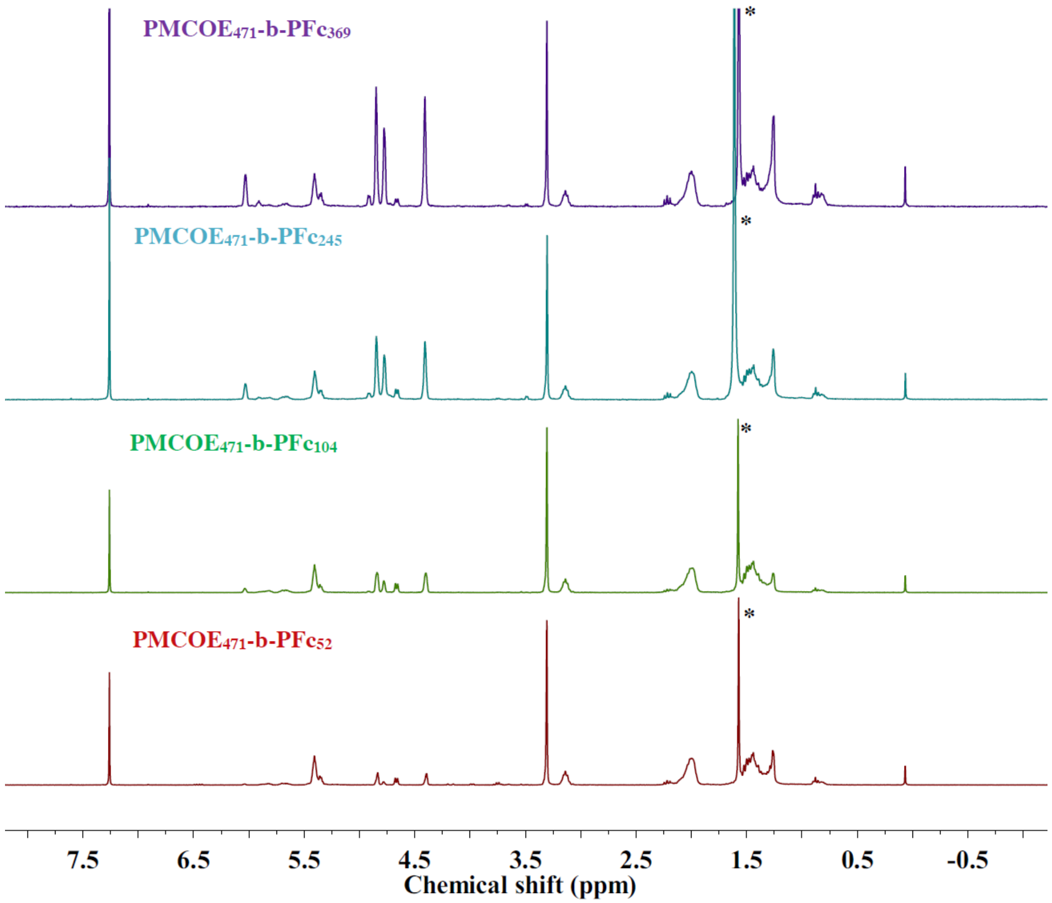

Figure S5. ^{1}H NMR ($CDCl_3$) spectra of PMCOE-*b*-PFc.

Table S2. Characterization data of $PMCO_{92}$-*b*-PFc_n by sequential ROMP with aliquots taken out at specific time intervals.

Sample	Reaction time	Obtained Block Ratio	Second block conversion[a]	BCP[b]	M_n (Da)[c]
5	1 min	10:0.5	5%	$PMCO_{92}$-b-PFc_{5}	14,500
6	2 min	10:0.77	7.7%	$PMCO_{92}$-b-PFc_{7}	15,200
7	5 min	10:1.41	14.1%	$PMCO_{92}$-b-PFc_{13}	17,100
8	10 min	10:2.36	23.6%	$PMCO_{92}$-b-PFc_{22}	20,100
9	15 min	10:3	30%	$PMCO_{92}$-b-PFc_{28}	22,000
10	30 min	10:5	50%	$PMCO_{92}$-b-PFc_{46}	27,900
11	60 min	10:6.6	66.2%	$PMCO_{92}$-b-PFc_{61}	32,800

[a]The conversion was determined from ^{1}H NMR spectra.

[b]The DP of the first block was determined from GPC, the DP of the second block was determined from ^{1}H NMR.

[c]The number molecular weight was calculated based on the DP of the diblock .

3.3 Synthesis of PNR-*b*-PFc

Scheme S2. Synthetic scheme towards block copolymer PNR-*b*-PFc.

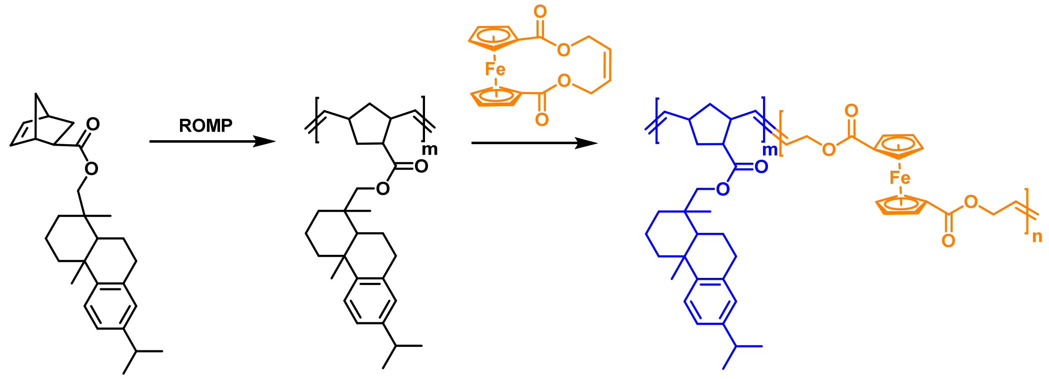

3.3.1 Synthesis of PNR$_{123}$-*b*-PFc$_{93}$

Dehydroabietic alcohol modified norbornene (0.46 mmol, 187 mg, 100 eq) was dissolved in 1.75 mL DCM. Grubbs III catalyst (3.343 mg, 0.0046 mmol, 1 eq) in 0.05 mL DCM was added into the above solution under vigorous stirring for 30 min at room temperature (^{1}H NMR analysis indicated the complete consumption of monomer). 0.4 mL Sample was withdrawn for GPC analysis (M_n = 49,900 Da, $Đ$ = 1.18). Then 1,1'-(2-butenyl)ferrocenedicarboxylate (0.368 mmol, 120 mg, 80 eq) was added into the system. The reaction was conducted at 25 °C for another 10 h and then quenched with several drops of EVE. The product mixture was dispersed in chloroform, precipitated into methanol three times and dried, yielding 180 mg solid.

3.3.2 Synthesis of PNR$_{123}$-*b*-PFc$_{36}$

Dehydroabietic alcohol modified norbornene (0.46 mmol, 187 mg, 100 eq) was dissolved in 1.75 mL DCM. Grubbs III catalyst (3.343 mg, 0.0046 mmol, 1 eq) in 0.05 mL DCM was added into the above solution under vigorous stirring for 30 min at room temperature (^{1}H NMR analysis indicated the complete consumption of monomer). 0.4 mL Sample was withdrawn for GPC analysis (M_n = 49,900 Da, $Đ$ = 1.18). Then 1,1'-(2-butenyl)ferrocenedicarboxylate (0.184 mmol, 60 mg, 40 eq) was added into the system. The reaction was conducted at 25 °C for another 10 h and then quenched with several drops of EVE. The product mixture was dispersed in chloroform, precipitated into methanol three times and dried, yielding 140 mg solid.

3.3.3 Synthesis of PNR$_{52}$-*b*-PFc$_{71}$

Dehydroabietic alcohol modified norbornene (0.368 mmol, 149.6 mg, 50 eq) was dissolved in 1.25 mL DCM, Grubbs catalyst III (2.674 mg, 0.00368 mmol, 1 eq) in 0.1 mL DCM was added into the above solution under vigorous stirring for 30 min at room temperature (^{1}H NMR analysis indicated the complete consumption of monomer). 0.4

mL Sample was withdrawn for GPC analysis (M_n = 21,300 Da, $Đ$ = 1.11). Then 1,1'-(2-butenyl)ferrocenedicarboxylate (0.4906 mmol, 160 mg, 66.7 eq) dissolved in 0.5 mL DCM was added into the system. The reaction was conducted at 25 °C for another 10 h and then quenched with several drops of EVE. The product mixture was dispersed in chloroform, precipitated into methanol three times and dried, yielding 170 mg solid.

3.3.4 Synthesis of PNR$_{52}$-*b*-PFc$_{43}$

Dehydroabietic alcohol modified norbornene (0.368 mmol, 149.6 mg, 50 eq) was dissolved in 1.25 mL DCM. Grubbs III catalyst (2.674 mg, 0.00368 mmol, 1 eq) in 0.1 mL DCM was added into the above solution under vigorous stirring for 30 min at room temperature (^{1}H NMR analysis indicated the complete consumption of monomer). 0.4 mL Sample was withdrawn for GPC analysis (M_n = 21,300 Da, $Đ$ = 1.11). Then 1,1'-(2-butenyl)ferrocenedicarboxylate (0.2453 mmol, 80 mg, 33.3 eq) was added into the system. The reaction was conducted at 25 °C for another 10 h and then quenched with several drops of EVE. The product mixture was dispersed in chloroform, precipitated into methanol three times and dried, yielding 120 mg solid.

3.4 Characterization of PNR-*b*-PFc

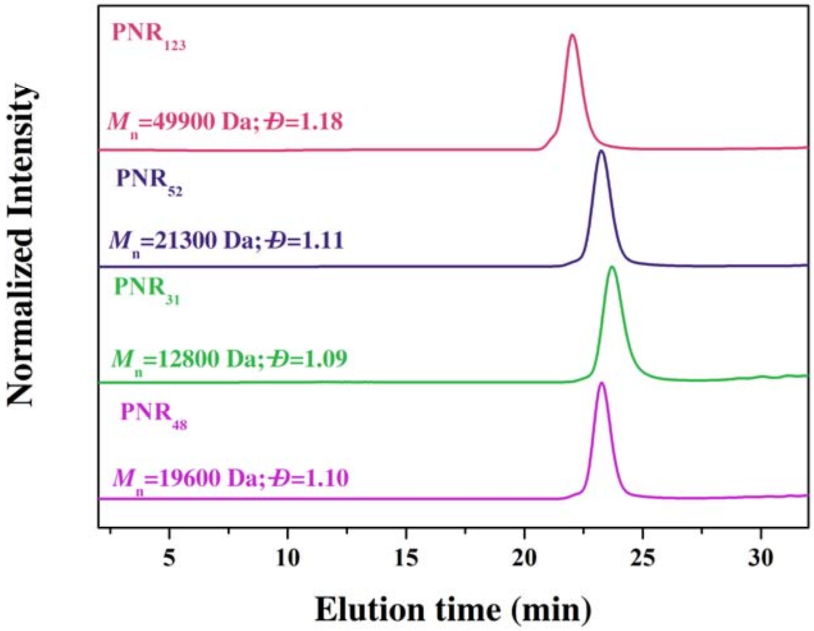

Figure S6. GPC traces of PNR with different molecular weight.

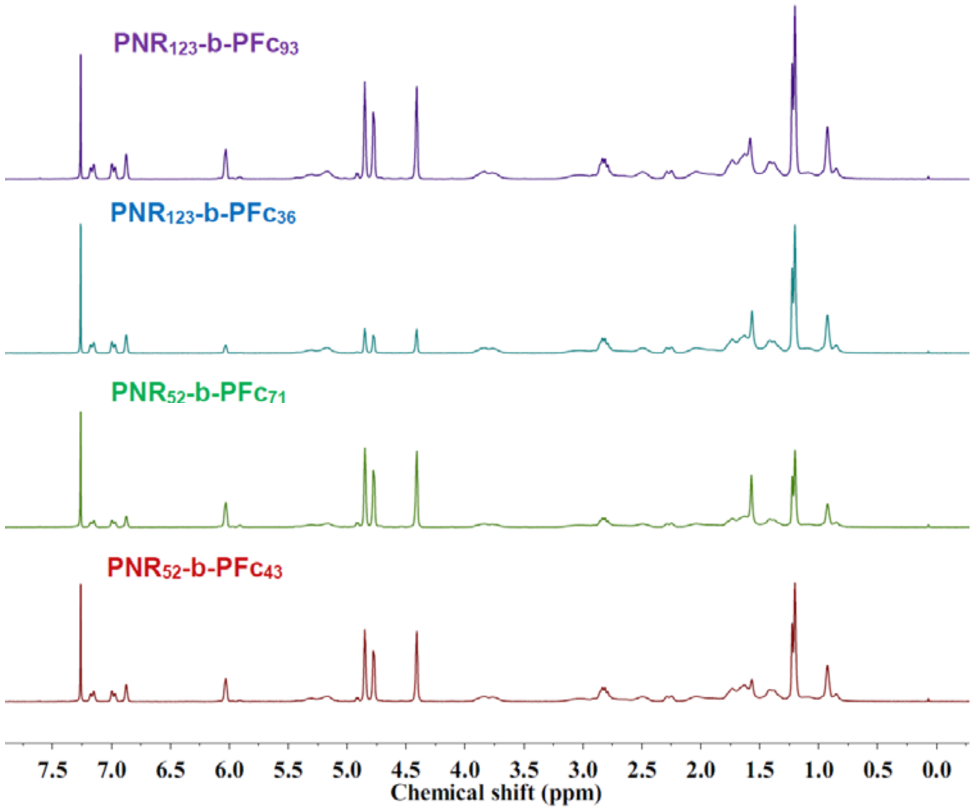

Figure S7. ^{1}H NMR ($CDCl_3$) spectra of block copolymer PNR-*b*-PFc.

3.5 Synthesis and PNR-*b*-PRc

Scheme S3. Synthetic scheme towards block copolymer PNR-*b*-PRc.

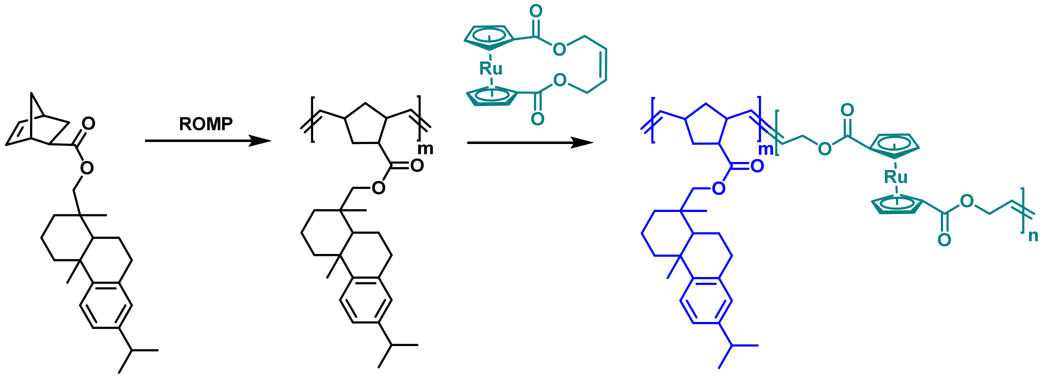

3.5.1 Synthesis of PNR$_{31}$-*b*-PRc$_{49}$

Dehydroabietic alcohol modified norbornene (0.368 mmol, 149.6 mg, 30 eq) was dissolved in 1.25 mL DCM. Grubbs III catalyst (8.915 mg, 0.01227 mmol, 1 eq) in 0.1 mL DCM was added into the above solution under vigorous stirring for 30 min at room temperature (^{1}H NMR analysis indicated the complete consumption of monomer). 0.4 mL Sample was withdrawn for GPC analysis (M_n = 12,800 Da, $Đ$ = 1.09). Then 1,1'-

(2-butenyl)ruthenocenedicarboxylate (0.4906 mmol, 182.2 mg, 40 eq) dissolved in 1 mL DCM was added into the system. The reaction was conducted at room temperature for another 10 h and then quenched with several drops of EVE. The product mixture was dispersed in chloroform, precipitated into methanol three times and dried, yielding 180 mg solid.

3.5.2 Synthesis of PNR_{31}-*b*-PRc_{21}

Dehydroabietic alcohol modified norbornene (0.368 mmol, 149.6 mg, 30 eq) was dissolved in 1.25 mL DCM. Grubbs III catalyst (8.915 mg, 0.01227 mmol, 1 eq) in 0.1 mL DCM was added into the above solution under vigorous stirring for 30 min at room temperature (^{1}H NMR analysis indicated the complete consumption of monomer). 0.4 mL Sample was withdrawn for GPC analysis (M_n = 12,800 Da, $Đ$ = 1.09). Then 1,1'-(2-butenyl)ruthenocenedicarboxylate (0.2453 mmol, 91.1 mg, 20 eq) was added into the system. The reaction was conducted at room temperature for another 10 h and then quenched with several drops of EVE. The product mixture was dispersed in chloroform, precipitated into methanol three times and dried, yielding 120 mg solid.

3.5.3 Synthesis of PNR_{48}-*b*-PRc_{56}

Dehydroabietic alcohol modified norbornene (0.368 mmol, 149.6 mg, 50 eq) was dissolved in 1.25 mL DCM, Grubbs III (5.35 mg, 0.00736 mmol, 1 eq) catalyst in 0.1 mL DCM was added into the above solution under vigorous stirring for 30 min at room temperature (^{1}H NMR analysis indicated the complete consumption of monomer). 0.4 mL Sample was withdrawn for GPC analysis (M_n = 19,700 Da, $Đ$ = 1.10). Then 1,1'-(2-butenyl)ruthenocenedicarboxylate (0.4906 mmol, 182.2 mg, 66.7 eq) dissolved in 1 mL DCM was added into the system. The reaction was conducted at room temperature for another 7 h and then quenched with several drops of EVE. The product mixture was dispersed in chloroform, precipitated into methanol three times and dried, yielding 170 mg solid.

3.5.3 Synthesis of PNR_{48}-*b*-PRc_n

Dehydroabietic alcohol modified norbornene (0.368 mmol, 149.6 mg, 50 eq) was dissolved in 1.25 mL DCM. Grubbs III catalyst (5.35 mg, 0.00736 mmol, 1 eq) in 0.1 mL DCM was added into the above solution under vigorous stirring for 30 min at room temperature (^{1}H NMR analysis indicated the complete consumption of monomer). 0.4 mL Sample was withdrawn for GPC analysis (M_n = 19,700 Da, $Đ$ = 1.10). Then 1,1'-(2-butenyl)ruthenocenedicarboxylate (0.4906 mmol, 182.2 mg, 66.7 eq) dissolved in 1 mL DCM was added into the system. Some aliquots were withdrawn at specified time intervals and quenched with EVE, then diluted for TEM measurements and ^{1}H NMR analysis to determine the conversion. The characterization data are summarized in Table S3.

3.6 Characterization of PNR-*b*-PRc.

Table S3. Characterization data of PNR_{48}-*b*-PRc_n by sequential ROMP with aliquots taken out at specific time intervals.

Sample	conv	M_n (Da)	DP_n
1 min	9%	22,900	PNR_{48}-*b*-PRc_9
5 min	19%	26,200	PNR_{48}-*b*-PRc_{18}
20 min	23%	27,700	PNR_{48}-*b*-PRc_{22}
60 min	34%	31,800	PNR_{48}-*b*-PRc_{33}
120 min	40%	33,600	PNR_{48}-*b*-PRc_{38}
240 min	54%	38,800	PNR_{48}-*b*-PRc_{52}
400 min	58%	40,300	PNR_{48}-*b*-PRc_{56}

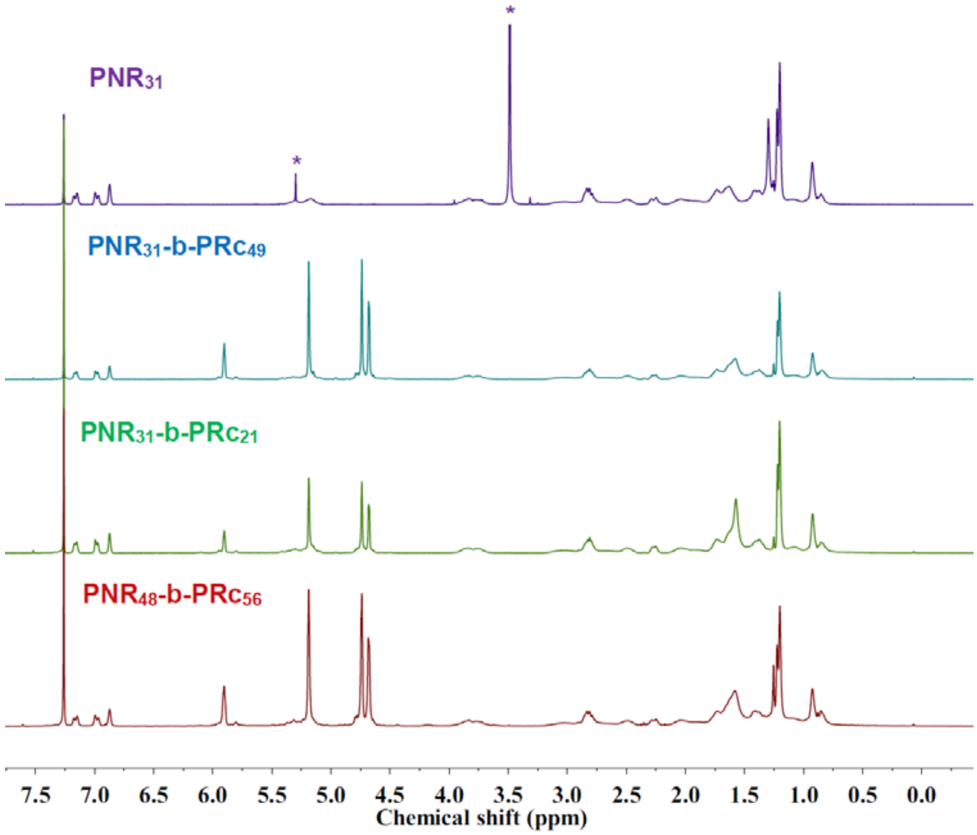

Figure S8. ^{1}H NMR ($CDCl_3$) spectra of ruthenocene-containing block copolymers.

4. ROMPI-CDSA of PMCOE-*b*-PFc

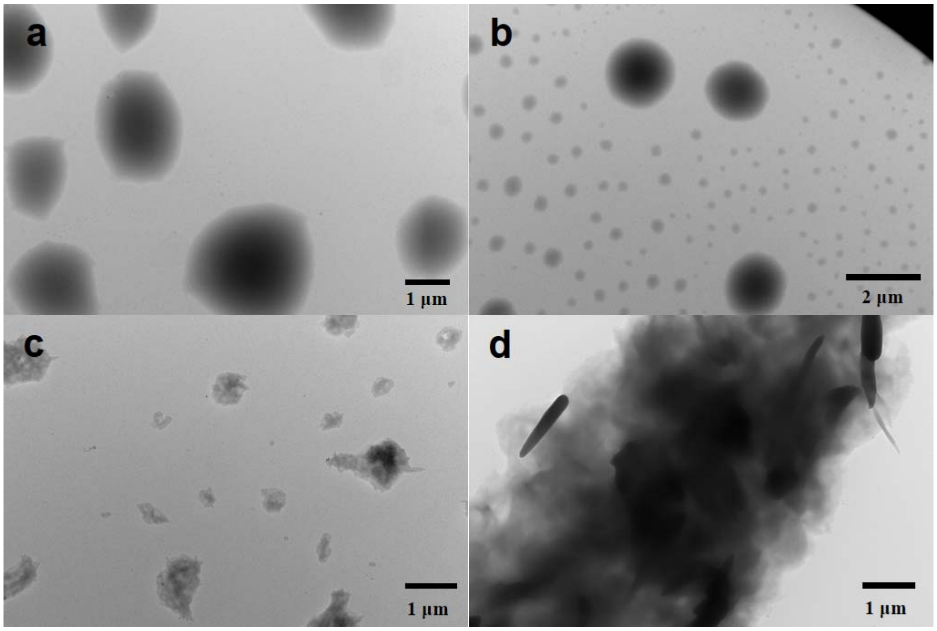

Figure S9. TEM images of the following ferrocene-containing block copolymers via ROMPI-CDSA: (a) $PMCOE_{471}$-*b*-PFc_{52}; (b) $PMCOE_{471}$-*b*-PFc_{104}; (c) $PMCOE_{471}$-*b*-PFc_{245}; (d) $PMCOE_{471}$-*b*-PFc_{369}.

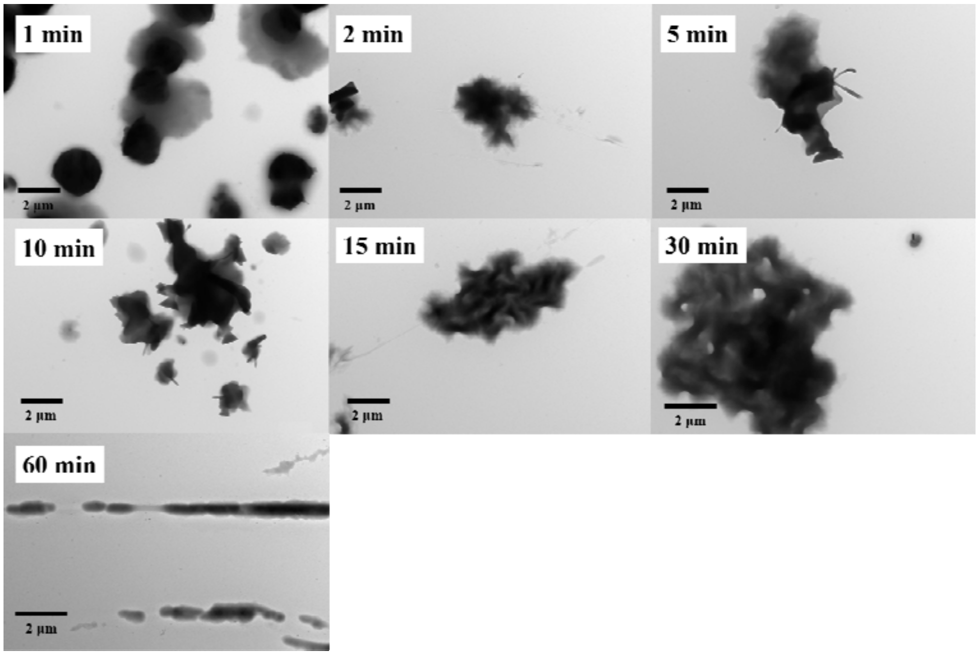

Figure S10. TEM images of block copolymer $PMCOE_{92}$-*b*-PFc_n via ROMPI-CDSA at different reaction time.

5. ROMPI-CDSA of PNR-*b*-PFc

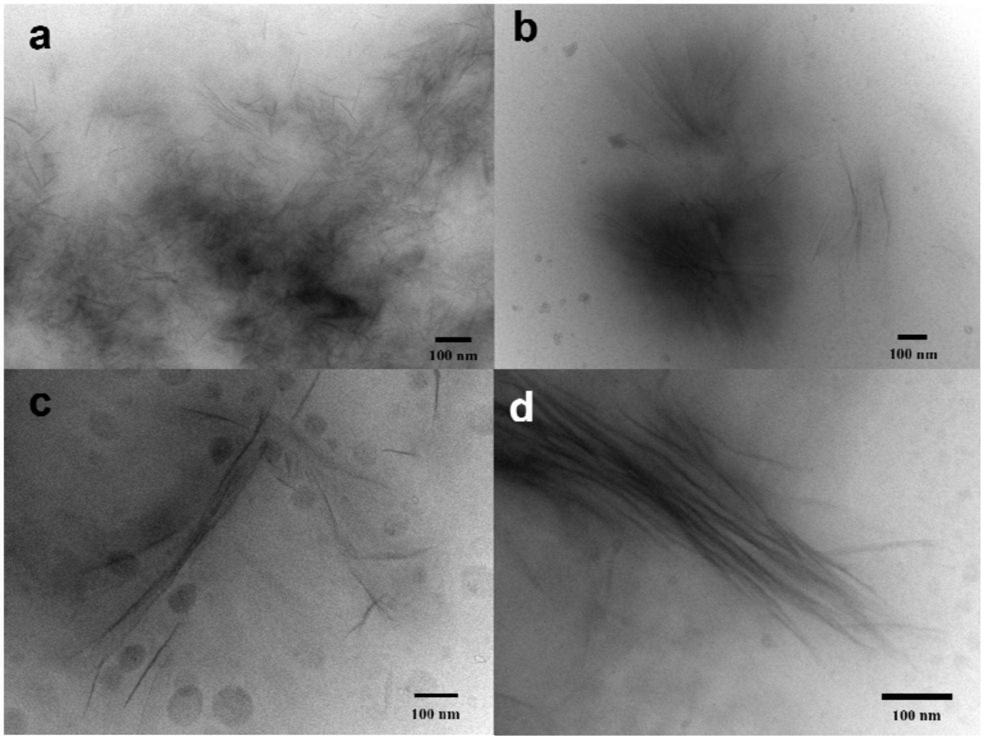

Figure S11. TEM images of the following ferrocene-containing block copolymers via ROMPI-CDSA: (a) PNR_{123}-*b*-PFc_{93}; (b) PNR_{123}-*b*-PFc_{36}; (c) PNR_{52}-*b*-PFc_{71}; (d) PNR_{52}-*b*-PFc_{43}.

6. Comparison of XRD spectra for RPc homopolymer and block copolymer

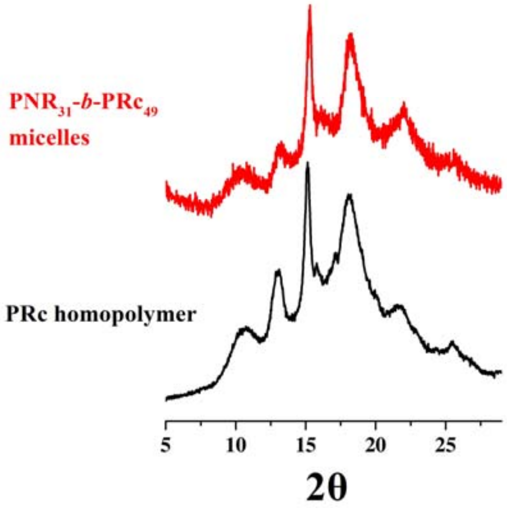

Figure S12. XRD spectra for films of PRc homopolymer and deposited micelles of PNR_{31}-*b*-PRc_{49}.

7. CDSA behavior of PNR_{31}-*b*-PRc_{49}

0.5 mg PNR_{31}-*b*-PRc_{49} was dissolved in 1 mL THF, carefully sealed and annealed in a preheated oven at 100 °C for 1 h, then the solution was cooled down to room temperature slowly (~5 h). The solution was cast onto copper grids for TEM analysis. The morphologies are shown below.

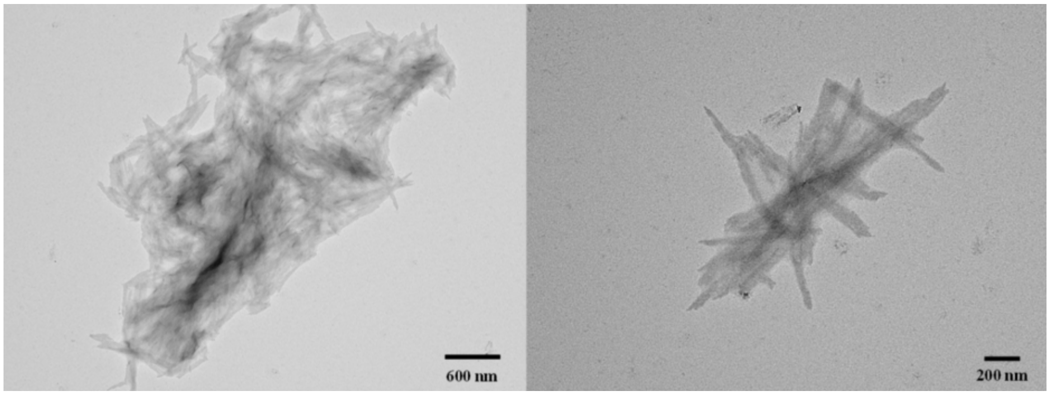

Figure S13. TEM images of ruthenocene-containing block copolymer PNR_{31}-*b*-PRc_{49} via CDSA.

8. Chain folding analysis of PNR_{31}-*b*-PRc_{49} micelles prepared via ROMPI-CDSA

The thickness of the crystalline core PRc in the platelets can be calculated based on the following equation proposed by Stephen Cheng[4]:

$$\begin{aligned} d_{PRu} &= d_{overall} \times \frac{M_n^{PRu} / \rho_{PRu}}{M_n^{PRu} / \rho_{PRu} + M_n^{PNR} / \rho_{PNR}} \\ &= 8.0 \text{ nm} \times \frac{18200/1.5}{18200/1.5 + 12800/1.07} \\ &= 4.0 \text{ nm} \end{aligned}$$

where $d_{overall}$ is the total thickness of the platelets, M_n^{PRu} is the molecular weight of PRc, M_n^{PNR} is the molecular weight of PNR, ρ_{PRu} is the density of PRc and ρ_{PNR} is the density of PNR.[3b] For simplification, the density of crystalline PRc and non-crystalline PRc was roughly considered to be same, measured through use of a density gradient column at room temperature.

Chain unit length L of PRc was calculated to be 1.263 nm, referenced from our previous work.[2c]

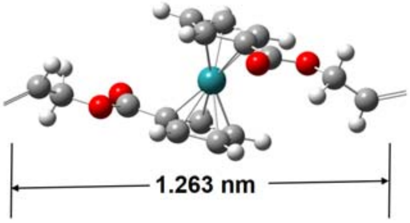

The quantity of chain units n in the platelets normal to the fold surface was calculated to be

$$n = \frac{d_{PRu}}{L} = \frac{4\ \text{nm}}{1.263\ \text{nm}} = 3.16 \approx 3$$

Then the chain folding times n_f was calculated as

$$n_f = \frac{DP}{n} = \frac{49}{3} = 16.3 \approx 16$$

9. ROMPI-CDSA of PNR_{31}-*b*-PRc_{21}

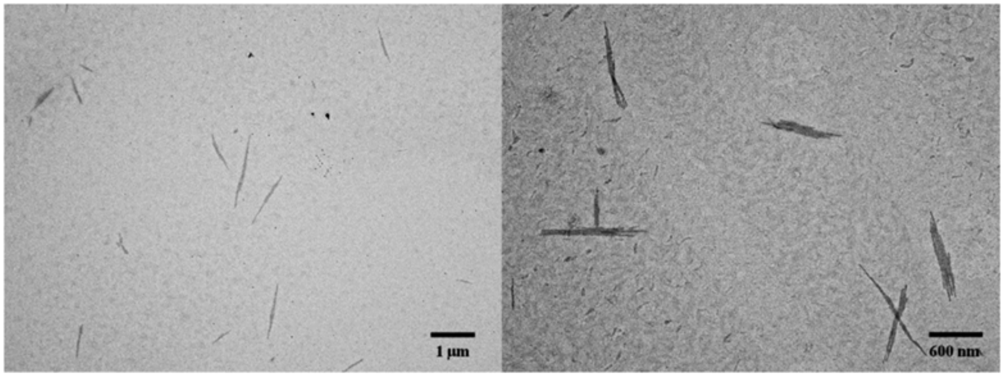

Figure S14. TEM images of ruthenocene-containing block copolymer PNR_{31}-*b*-PRc_{21} via ROMPI-CDSA.

10. ROMPI-CDSA of PNR_{48}-*b*-PRc_{56}

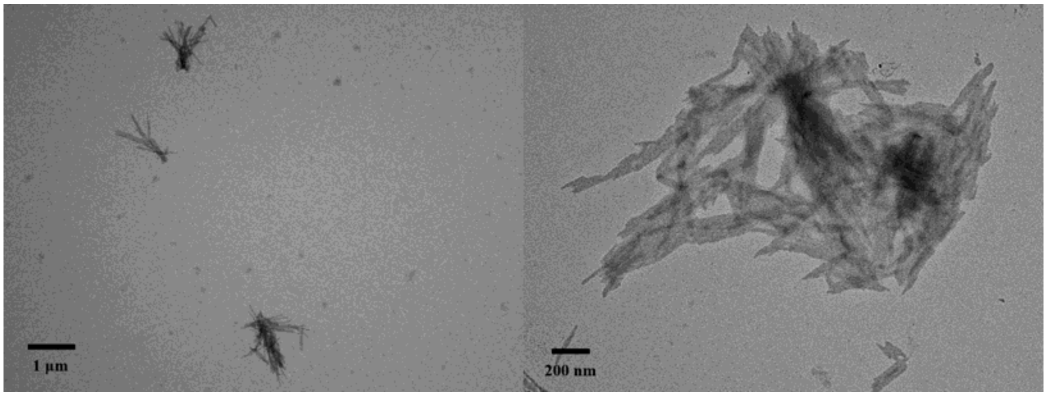

Figure S15. TEM images of ruthenocene-containing block copolymer PNR_{48}-*b*-PRc_{56} via ROMPI-CDSA.

11. ROMPI-CDSA of PNR_{48}-*b*-PRc_n

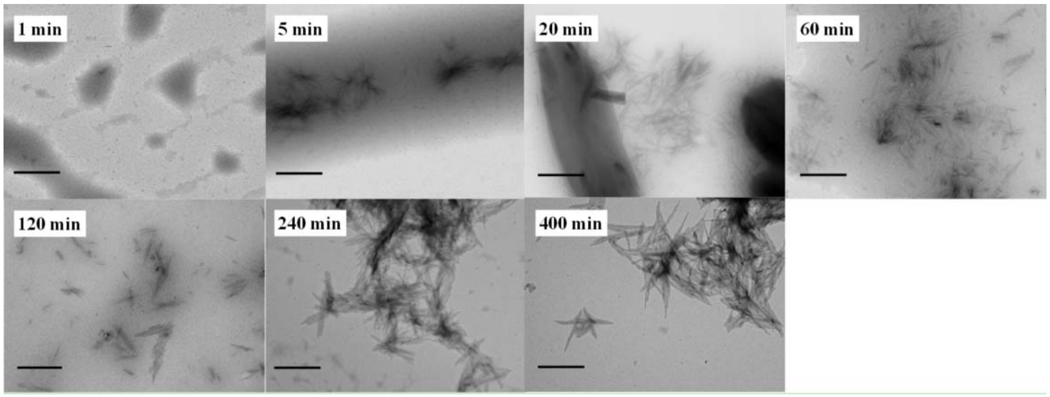

Figure S16. Morphological transition from unimer films to bundled fibers and then to platelets. Low magnification TEM images of PNR_{48}-*b*-PRc_n micelles via ROMPI-CDSA at different time. The scale bar is 1 μm.

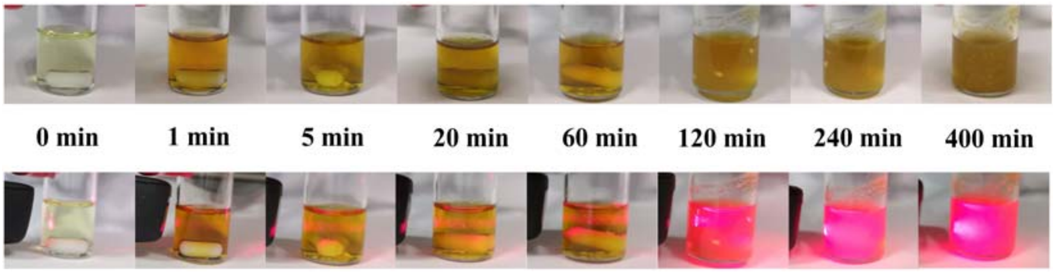

Figure S17. Representative pictures of PNR_{48}-*b*-PRc_n micelles by ROMPI-CDSA at specific time intervals. Upper image: snapshots of reaction vial; Lower image: snapshots of reaction vial with a laser beam to show the Tyndall effect.

12. Comparison of SAED patterns of PNR_{31}-*b*-PRc_{49} and $PMCOE_{471}$-*b*-PFc_{52}.

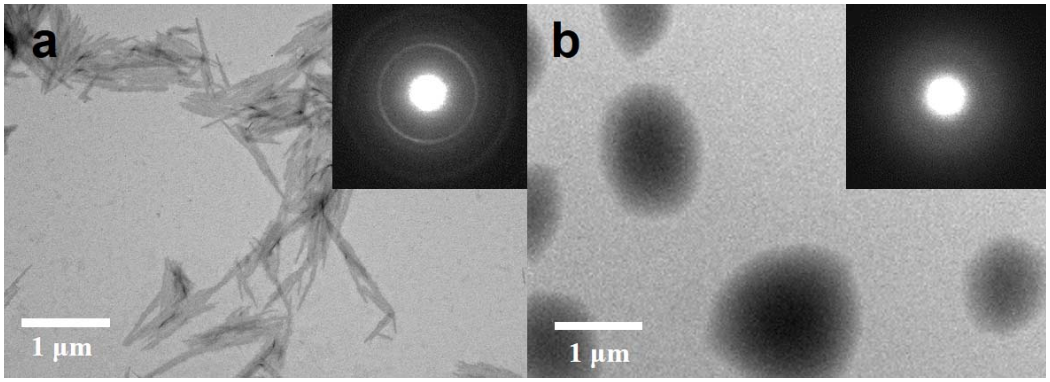

Figure S18. TEM images of (a) PNR_{31}-*b*-PRc_{49} and (b) $PMCOE_{471}$-*b*-PFc_{52} micelles formed during polymerization. The inset images show the SAED patterns.

12. References

[1] M. S. Sanford, J. A. Love, R. H. Grubbs, *Organometallics* **2001**, *20*, 5314-5318.

[2] a) Y. Sha, Y. Zhang, E. Xu, Z. Wang, T. Zhu, S. L. Craig, C. Tang, *ACS Macro Lett* **2018**, *7*, 1174-1179; b) Y. Sha, Y. Zhang, T. Zhu, S. Tan, Y. Cha, S. L. Craig, C. Tang, *Macromolecules* **2018**, *51*, 9131-9139; c) Y. Sha, Y. Zhang, E. Xu, C. W. McAlister, T. Zhu, S. L. Craig, C. Tang, *Chem. Sci.* **2019**, *10*, 4959-4965.

[3] a) M. A. Rahman, H. N. Lokupitiya, M. S. Ganewatta, L. Yuan, M. Stefik, C. Tang, *Macromolecules* **2017**, *50*, 2069-2077; b) M. S. Ganewatta, W. Ding, M. A. Rahman, L. Yuan, Z. Wang, N. Hamidi, M. L. Robertson, C. Tang, *Macromolecules* **2016**, *49*, 7155-7164.

[4] W. Chen, J. Zheng, S. Z. D. Cheng, C. Li, P. Huang, L. Zhu, H. Xiong, Q. Ge, Y. Guo, R. P. Quirk, B. Lotz, L. Deng, C. Wu, E. L. Thomas, *Phys. Rev. Lett.* **2004**, *93*, 028301.
